# Characteristics of an Extended Gate Field-Effect Transistor for Glucose Sensing Using an Enzyme-Containing Silk Fibroin Membrane as the Bio-Chemical Component

**DOI:** 10.3390/bios10060057

**Published:** 2020-05-29

**Authors:** Kazuto Koike, Taihou Sasaki, Kenta Hiraki, Kodai Ike, Yuichi Hirofuji, Mitsuaki Yano

**Affiliations:** Nanomaterials Microdevices Research Center, Osaka Institute of Technology, Asahi-ku Ohmiya, Osaka 535-8585, Japan; kazuto.koike@oit.ac.jp (K.K.); m1m19316@oit.ac.jp (T.S.); m1m20313@oit.ac.jp (K.H.); ike528123@gmail.com (K.I.); yhirofuji@aol.com (Y.H.)

**Keywords:** biosensor, EGFET, silk fibroin, glucose oxidase, immobilization

## Abstract

The characteristics of a glucose sensor based on an ion-sensitive TiO_2_/Ti extended gate electrode field-effect transistor (EGFET) are reported. A glucose oxidase-containing silk fibroin membrane was immobilized on a TiO_2_/Ti surface as the bio-sensing component. This EGFET-type biosensor was estimated to be able to detect a glucose concentration as low as 0.001 mg/mL in an aqueous electrolyte, which enables the sensing of glucose in the saliva and sweat. The endurance of this sensor was also examined, and it was found that the retention time of the original sensitivity for repeated use at room temperature was more than 30 days, with a high heat tolerance temperature close to 60 °C.

## 1. Introduction

Diabetes mellitus is a polygenic disease characterized by abnormally high β-D glucose levels in the blood and might cause disease complications such as visual disturbance, renal dysfunction, and angiopathy. To avoid falling into serious diabetes, it is important to control the glucose level by monitoring the daily concentration. To detect the glucose concentration in the blood, an enzymatic reaction is utilized, and portability with easy operation is required for sensors for personal use.

Most of the portable glucose sensors currently used are electrode-type, the initial study of which was based on an oxygen electrode [1]. In 1962, Clark et al. [2] succeeded in detecting the glucose in the blood by covering the oxygen electrode with glucose oxidase (GOD). Since glucose produces gluconic acid and hydrogen peroxide in the presence of GOD and oxygen, the glucose concentration was measured by the decrease in oxygen in the analyte solution. In 1973, Guilbault et al. [3] modified this approach to monitor the current flow by the oxidation of hydrogen peroxide on the electrode, and most glucose sensors today are based on this amperometric method.

Soon after this work by Guilbault et al., studies on potentiometric pH sensors using solution gate field-effect transistors (SGFETs) named ion-sensitive FETs (ISFETs) were started by many researchers [4,5]. The advantages of potentiometric measurement by FETs are as follows:

(1) The capability of the direct detection of the potential difference between the electrolyte and gate electrode due to the high input impedance of FETs, which enables continuous measurement free from electrolysis reactions.
(2) The capability of rapid measurement due to the simple detection mechanism and small sensor size, which enables stress-free operation.
(3) The capability of integration on a chip, which enables application to μ-total analysis systems.

The first potentiometric biosensor using ISFET was reported in 1980 by Caras et al. [6]. They developed a penicillin sensor by immobilizing penicillinase on the solution gate electrode of an ISFET. After that, ISFET-type biosensors such as immuno-sensors [7], urea sensors [8], neutral lipid sensors [9], and glucose sensors [10] were reported. These potentiometric biosensors employed Si-based ISFETs; however, other ISFETs based on diamond [11], graphene [12,13], and AlGaN/GaN [14] have been applied recently to exploit relevant advantages. We also reported immuno-sensors [15] and glucose sensors [15,16] using ZnO-based ISFETs on glass substrates to exploit advantages such as transparency and stable operation under visible illumination.

Apart from these ISFET-type biosensors, Spiegel et al. [17] developed in 1983 a new FET-type ion sensor named extended gate FETs (EGFETs) by connecting an ion-sensitive extended electrode to the gated electrode of a commercially available metal-oxide-semiconductor FET (MOSFET). Since EGFETs do not require a specialized solution gate like ISFETs and can be used repeatedly by replacing the extended gate electrodes, their application to biosensors [18,19,20,21] is a current study of interest.

Both ISFET-type and EGFET-type glucose sensors using GOD detect the protons yielded by the following enzymatic reaction (1) between glucose and GOD.

(1) $$\beta \text{-}D\;\mathrm{glucose}+O_{2}+H_{2}O\;\overset{\mathrm{GOD}}{\Rightarrow }\;\mathrm{gluconolactone}+H_{2}O_{2}\Leftrightarrow \text{gluconic acid}+H^{+}.$$

When the protons are accepted by the receptors on gate electrode, the electrical potential of the gate electrode will be changed. Amino functions immobilized on the gate electrode and/or metal-oxide gate insulators themselves are used for the proton receptors.

To immobilize GOD on the ion-sensitive gate electrode, ionic binding, covalent binding, and inclusion methods have been developed. Among them, the covalent bonding method is mostly used for ISFET-type and EGFET-type biosensors, similar to the cases of our ZnO-based ISFET-type [15,16] and EGFET-type glucose sensors [21]. Although the number is limited, however, the inclusion method is also applied as reported by You et al. [22]. They immobilized an approximately 10 µm thick GOD-containing silk fibroin (SF) membrane on a graphene-based ISFET by using a casting method and succeeded in detecting glucose in the concentration range of 0.1 to 10 mM (0.018 to 1.8 mg/mL).

On the other hand, GOD-containing thick SF membranes have been applied to amperometric glucose sensors [23,24,25,26] and known to have advantages such as an increase in the GOD holding power in water [25,26] and the heat tolerance of enzymatic activity [24,26]. 

SF is a kind of protein in silk yarn and has been utilized for skincare cosmetics, medical supplies, and foodstuffs as a biocompatible material. The merits of SF for enzyme scaffold application are the ability of covalent bonding with enzyme molecules and the quantitative controllability of the amount of immobilized enzyme through the mixing ratio with SF. The SF membranes used in previous research work, however, were obtained from SF solutions extracted by the researchers themselves from the living silkworm or silk yarn and might have quality variation depending on the production areas, seasons, and skill levels of the operators.

In the present work, we report the characteristics of an EGFET-type glucose sensor, the GOD-containing SF membrane of which was prepared by a spin-coating method using an aqueous solution of commercially available high quality SF powder [27] mixed with GOD powder. To our knowledge, EGFET-type glucose sensors combined with GOD-containing SF membranes have not been reported. In addition, the development of the spin-coating method for this purpose enables the preparation of highly water-resistive SF membranes as thin as several µm thick with controlled thickness [28], and the use of the SF powder, which can withstand long-term storage of two years [29], enables the preparation of aqueous solutions at arbitrary concentrations without quality variation [28]. The major purpose of this work is to provide a reliable and cost-effective fabrication method for potentiometric glucose sensors for personal use.

## 2. Materials and Methods

### 2.1. Chemicals

High quality SF powder (CAS: 9009-99-8) [29] was supplied from Matsuda silk farm [29] (Tokyo, Japan) with the registered name “Nanofibroin Powder^®^”. Phosphate-buffered saline (PBS) reagents (CAS: 169-27185, 168-27155, 167-26125), sodium dihydrogenphosphate dehydrate (CAS: 13472-35-0), β-D glucose (CAS: 492-61-5), gluconolactone (CAS: 90-80-2), and gluconic acid (CAS: 526-95-4) were purchased from FUJIFILM Wako (Tokyo, Japan). *N*-2-(aminoethyl)-8-aminooctyltrimethoxysilane (AOTMS) was supplied from Shin-Etsu (Tokyo, Japan) with the article number KBM-6803. GOD powder (CAS: 9001-37-0) was purchased from Oriental East (Tokyo, Japan). A 0.2 mm-thick Ti (99.5%) plate used for the extended gate electrode was purchased from Nilaco (Tokyo, Japan) with the article number TI-453382.

### 2.2. Experimental Apparatus

The X-ray diffraction (XRD) measurement for TiO_2_/Ti was performed by using a SmartLab XRD system (Rigaku, Tokyo, Japan). The thickness of the TiO_2_ on the Ti substrate was measured by using a SE-2000 spectroscopic ellipsometer (Semilab Japan, Tokyo, Japan). The EGFET in this study was assembled by using a 2N7000 N-channel enhancement-type MOSFET (ON Semiconductor, Phoenix, AZ, USA). The pH and glucose measurements of our EGFETs were performed by using an RE-1B Ag/AgCl reference electrode (ALS, Tokyo, Japan) and a B1500A semiconductor device analyzer (Keysight, Tokyo, Japan). The pH change in the PBS during measurement was monitored by using an AT-610ST titrator equipped with a C171 glass electrode (Kyoto Electronics, Kyoto, Japan). The thickness, roughness, optical absorption, and surface morphology of the SF membrane were measured by using a DektakXTS-0K1704 stylus-type step profiler (Bruker Japan, Kanagawa, Japan), an SPM9700 atomic force microscope (AFM) (Shimazu, Kyoto, Japan), an FTIR-8400S Fourier transform infrared spectrometer (FTIR) with a DRS-8000A diffuse reflector (Shimazu, Kyoto, Japan), and a VE8800 scanning electron microscope (SEM) (Keyence, Osaka, Japan), respectively.

### 2.3. Device Setup and Measuring Conditions Used for pH and Glucose Sensing

**Figure 1:**
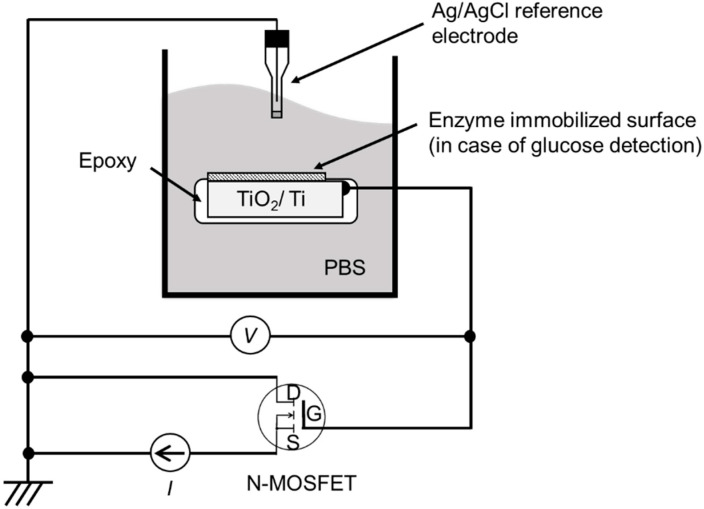
Device setup used for the measurement of ion sensitivity and glucose sensitivity [21]. The TiO_2_/Ti plate in PBS and the N-channel enhancement-type MOSFET in air construct the extended gate electrode field-effect transistor (EGFET).

To check the sensitivity of the EGFET-type ion/glucose sensors, the experimental setup shown in Figure 1 [21] was employed, where the electrolyte was 100 mM PBS. The extended gate electrode was immersed in the electrolyte and connected with the gate electrode of an outside MOSFET. To insulate against the electrolyte, the extended gate electrode was molded with epoxy resin except the sensing area of about 4 mm × 4 mm on the surface. The pH/glucose concentration dependence of the gate electrode potential, Δ*V*, was measured as the voltage shift of the drain bias to the Ag/AgCl reference electrode by maintaining the source-drain current at a constant value of 100 μA in the saturation region (ΔI/ΔV≅0). To monitor the pH change at an offshore point during the measurement, a glass electrode of a pH meter was separately located in the electrolyte although is not shown in Figure 1.

To measure the pH sensitivity of the EGFET-type ion sensors, the electrolyte was kept at 25 °C; the pH was controlled at 6.1, 6.6, 7.0, and 7.5 [21] by changing the mixing ratio of the commercially available PBS reagents at pH = 8.0, 7.0, and 6.0; and the pH was controlled at 5.5 by mixing sodium dihydrogenphosphate dehydrate with the PBS reagent at pH = 6.0. To measure the glucose sensitivity, on the other hand, a specific quantity of β-D glucose powder was dissolved in 100 mM PBS at pH = 6.0, and the solution during the measurement was kept at 37 °C to activate the enzymatic function at around the optimal condition.

### 2.4. Modification Process for the TiO_2_ Surface and Preparation Process for the Silk Fibroin Membranes

The surface modification of the TiO_2_/Ti extended gate electrode was performed by the following silanization process using a silane coupling treatment [21,28]:

(1) Purify the TiO_2_ surface by ultrasonic washing in organic solvent followed by UV ozone cleaning.
(2) Immerse in 2 vol % AOTMS solution in 2-propanol solvent at 35 °C and shake for 30 min.
(3) Bake in air at 120 °C for 30 min.

The SF membranes on the TiO_2_/Ti extended gate electrodes and glass substrates were prepared by the following wet process using a spin-coating method [28]:

(1) Dissolve SF powder in deionized water with a specific ratio (0–40 wt %; it becomes difficult to drip at concentrations higher than 50 wt % due to the increase in viscosity with concentration).
(2) Add a specific quantity of GOD powder (1–2 wt %) in the SF aqueous solution and stir at room temperature.
(3) Apply the GOD-containing SF aqueous solution to the substrate surface by a spin-coating method.
(4) Insolubilize the coated layer by immersing into 80 vol % ethanol aqueous solution at room temperature for 1 h.
(5) Dry in air at room temperature after rinsing in deionized water.

## 3. Results and Discussion

### 3.1. Preparation of EGFET-Type Ion Sensor

**Figure 2:**
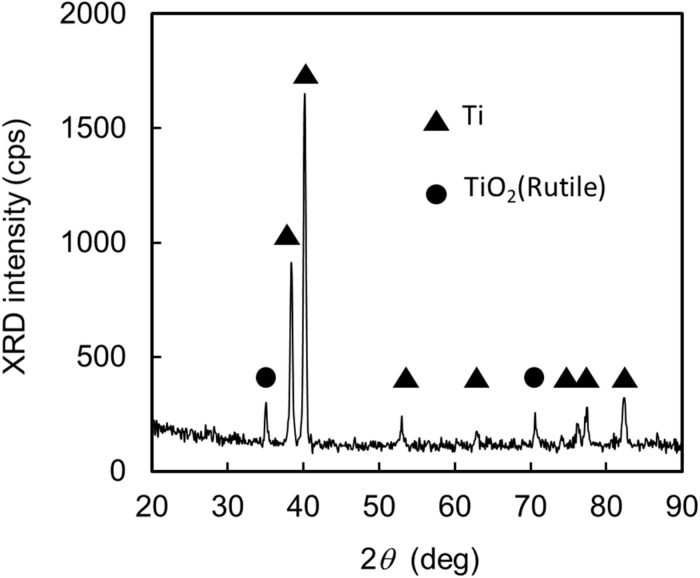
Grazing-incident X-ray diffraction (GIXRD) pattern of the Ti plate surface after heat treatment in O_2_ at 500 °C for 10 min. The assignment of the respective peaks is given in the figure.

TiO_2_ is known to have excellent pH sensitivity and has been applied to the proton receptors of FET-type pH sensors [30,31,32,33]. In this study, we employed a thin TiO_2_ layer on a metallic Ti plate for the ion-sensitive extended gate electrode, the Ti plate of which was 5 mm wide, 6 mm long, and 0.2 mm thick in dimension [21]. After ultrasonic washing in organic solvent followed by UV ozone cleaning, the plate was heated in an O_2_ atmosphere at 500 °C for 10 min to form the oxidized layer as the proton receptor. Figure 2 shows the grazing-incident X-ray diffraction (GIXRD) pattern of the Ti plate after oxidization. Clear peaks from rutile TiO_2_ are seen in addition to those from Ti. The thickness of the TiO_2_ layer estimated by spectroscopic ellipsometry was approximately 7 nm. This TiO_2_/Ti plate was used for the extended gate electrode and connected to the gate electrode of an N-channel enhancement-type MOSFET to construct an EGFET-type ion sensor.

**Figure 3:**
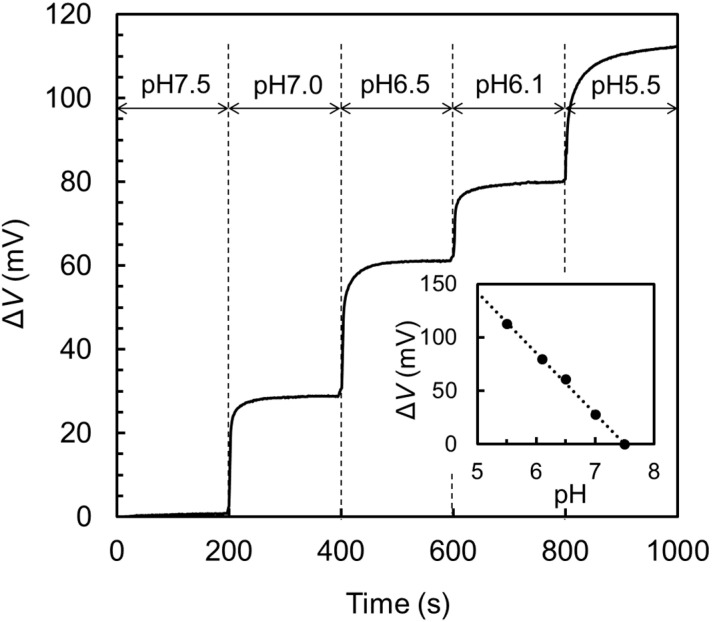
pH response of the TiO_2_/Ti extended electrode at 25 °C.

Figure 3 shows the response of the gate electrode potential, Δ*V*, to the step-like pH decrease of 7.5 → 7.0 → 6.5 → 6.1 → 5.5. The response was exponential-like with a time constant of several seconds, suggesting that the response can be expressed by a first order equilibrium reaction. The inset shows the saturated values of the Δ*V* at the respective pH steps. The slope of the straight line in the inset is 57 mV/pH which roughly agrees with the theoretical value derived from the Nernst equation, 59 mV/pH [34], at room temperature. From these results, it is clearly understood that the TiO_2_ layer on the Ti plate works as an ideal proton receptor.

### 3.2. Formation of a GOD-Containing SF Membrane on the Extended Gate Electrode

In the next step, a GOD-containing SF membrane was formed on the TiO_2_/Ti ion-sensitive extended gate electrode to fabricate the EGFET-type glucose sensor. The preparation process for the GOD-containing SF membrane has been given in the previous section.

The resulting membrane was transparent and stable in air; however, the adhesivity to the TiO_2_ surface was not enough when directly formed on the surface; it was easily removed from the TiO_2_/Ti extended gate electrode by the rinsing in deionized water and the measurement in PBS. To address this difficulty, it was effective to treat the TiO_2_ surface in advance with a long-chain aminosilane coupling agent. We used AOTMS as the coupling agent and modified the TiO_2_ surface by using the silanization process described in the previous section. This modification of the TiO_2_ surface dramatically improved the water resistance against removal; the SF membrane was mechanically stable, even in water, and did not remove from the gate electrode at all after 1 weak of immersion in PBS followed by 10 min of ultrasonic washing in deionized water. This improvement is caused by the formation of amide bonds between the NH_2_-terminal groups of AOTMS and the carboxyl groups in SF [28].

**Figure 4:**
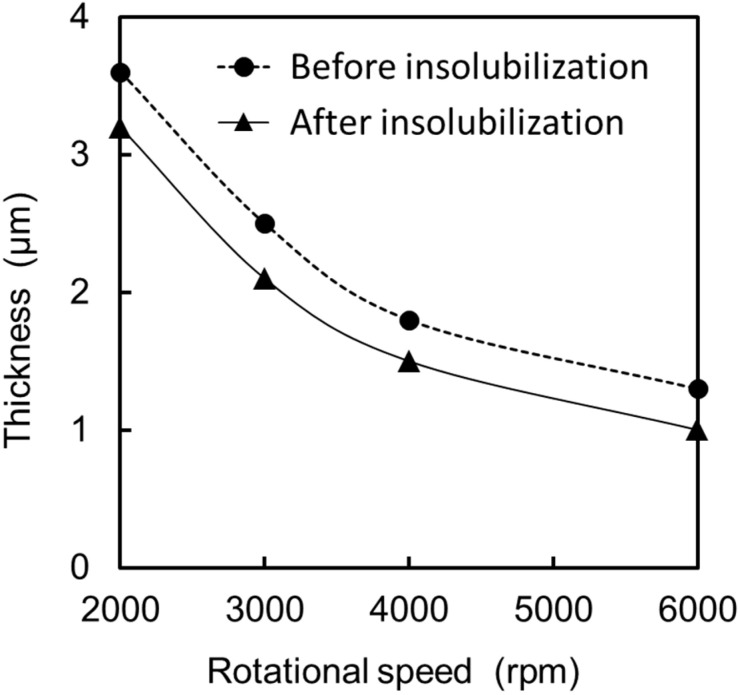
Relationship between the rotational speed during spin coating and the thickness of the silk fibroin (SF) membranes before and after insolubilization. The SF concentration in the aqueous solution used for the spin-coating was 30 wt %.

Figure 4 shows the relationship between the rotational speed during spin coating and the thickness of the SF membrane before and after insolubilization. These membranes were formed on glass substrates using a 30 wt % SF aqueous solution, and the sample, before insolubilization, was prepared by drying in air after the spin coating. The thickness was measured with a stylus-type step profiler. We repeated this experiment three times and found that the reproducibility was within the error of ±5%. These experimental results indicate that the thickness of the SF membrane can be controlled by the rotational speed during spin coating. Although the experimental data are not shown, the thickness was also controllable by the concentration of the SF aqueous solution, since its viscosity increased with concentration [28].

The root mean square roughness of the surface measured by AFM was about 0.23 nm and 2.2 nm for the membranes before and after insolubilization, respectively [28]. It is shown in Figure 4 that the thickness was reduced by about 23% after the insolubilization. Part of this reduction was due to elution to the ethanol aqueous solution during insolubilization, and another part was due to the partial crystallization of the SF by a dehydration reaction.

It is known in general that there are three secondary structural components in SF; random coils (amorphous), α-helices (crystalline), and β-pleated sheets (crystalline). It is also known that the random coil structure is changed to the β-pleated sheet structure by the insolubilization using an ethanol aqueous solution [35,36]. To our SF membranes, we analyzed the structural change by measuring infrared absorption spectra using FTIR and confirmed that the spectrum before insolubilization was dominated by the peaks related to the random coil structure, and that after insolubilization was dominated by the peaks related to the β-pleated sheet structure [28].

**Figure 5:**
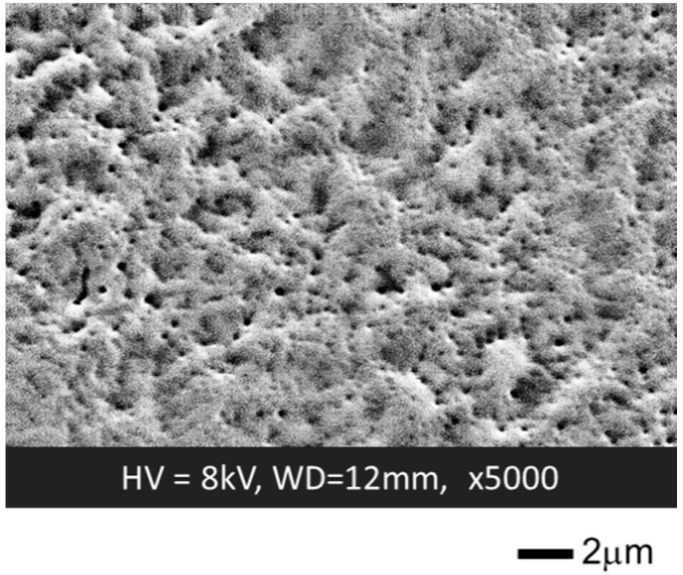
SEM image of the surface of an SF membrane after insolubilization [28].

Figure 5 depicts an SEM image of the SF membrane surface after insolubilization [28]. It is seen that the membrane has a textured structure with many nano-size holes less than 1 µm in diameter. Such a nanoporous structure was also observed for the SF membranes prepared from live silkworms and considered to result from the simultaneous progress of the elution and crystallization during insolubilization. From the viewpoint of the application to biosensors, this nanoporous structure is favorable to increase the specific surface area for the enzymatic reaction and help the proton transport between the TiO_2_ surface and outside of the membrane.

### 3.3. Characteristics of a Glucose Sensor

In this section, the GOD-containing SF membrane was prepared by using a weight ratio of GOD/SF/H_2_O of 1.5:30:70, and the solution was spin-coated on the TiO_2_/Ti ion-sensitive extended gate electrode at 6000 rpm. The thickness of the GOD-containing SF membrane after insolubilization was approximately 1 µm.

**Figure 6:**
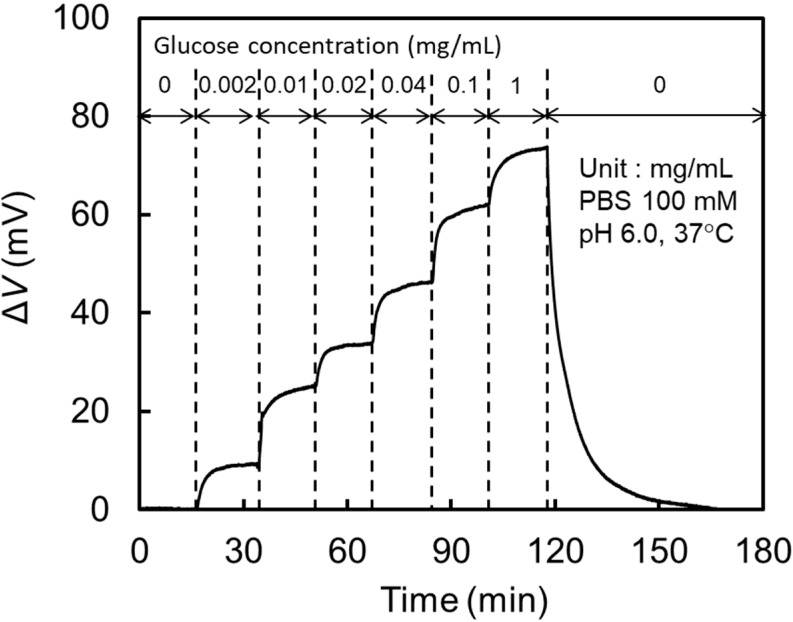
Glucose response of the EGFET-type biosensor at pH = 6.0 and 37 °C.

Figure 6 shows the response of Δ*V* to the step-like sequential change in the glucose concentration in PBS, 0 → 0.002 → 0.01 → 0.02 → 0.04 → 0.1 → 1 → 0 mg/mL. Since Δ*V* was increased with the increase in glucose concentration, it is clearly understood that protons yielded by the enzymatic reaction (1) were effectively transported to the ion-sensitive TiO_2_ surface on the extended gate electrode. The reversibility of the reaction can be confirmed by the recovery to Δ*V* = 0 V under the 1 → 0 mg/mL operation. During the measurement, the pH at an offshore point in PBS measured by a pH meter with the glass electrode did not show any change within the detection limit of 0.01 pH.

**Figure 7:**
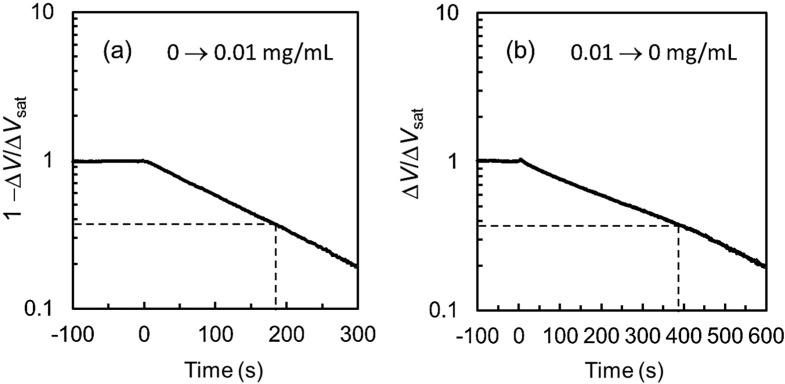
Response curves of Δ*V* at pH = 6.0 and 37 °C with a step-like increase (**a**) and decrease (**b**) in the glucose concentration in PBS.

Figure 7 shows the Δ*V* response to the step-like changes in glucose concentration from 0 to 0.01 mg/mL (a) and from 0.01 to 0 mg/mL (b). In both figures, the vertical axes are given in logarithmic scales and normalized by the change in the gate electrode potential at saturation, Δ*V*_sat_, and the response in (a) is depicted by (1 − Δ*V*)/Δ*V*_sat_. The time constant of the exponential-like curve in Figure 7a for the increase in glucose concentration is 180 s, and that in Figure 7b for the decrease in glucose concentration is 390 s. These values are roughly two-orders of magnitude larger than those of the response to pH changes in Figure 3.

This difference is presumably due to the hindering of proton transport by the SF membrane and/or the existence of a rate-limiting step in the proton generation reaction given by the enzymatic reaction (1). To study the slow response, we examined the detection speed at pH = 6.0 and 37 °C for the solutions of gluconolactone in PBS and gluconic acid in PBS, and found that the response time constant for gluconolactone detection was several hundred seconds, while that for gluconic acid was approximately 10 s. From these results, we consider at present that the hydrolysis reaction for gluconolactone might be the rate-limiting step in the slow glucose detection, although further study must be done.

Subsequently, we analyzed the Δ*V* response in Figure 6 using a Michaelis–Menten equation [37],

(2) $$\Delta V=\frac{\Delta V_{\max}C_{\mathrm{gluc}}}{K_{m}^{\mathrm{app}}+C_{\mathrm{gluc}}},$$

where $C_{\mathrm{gluc}}$ is the glucose concentration in PBS, $\Delta V_{\max}$ is the maximum change in the gate electrode potential, and $K_{m}^{\mathrm{app}}$ is the apparent Michaelis constant. The values of $\Delta V_{\max}$ and $K_{m}^{\mathrm{app}}$ can be estimated from the experimental data by rearranging Equation (2) into Equation (3) and adopting the experimental data in a Hanes–Woolf plot [38],

(3) $$\frac{C_{\mathrm{gluc}}}{\Delta V}=\frac{C_{\mathrm{gluc}}}{\Delta V_{\max}}+\frac{K_{m}^{\mathrm{app}}}{\Delta V_{\max}}.$$

**Figure 8:**
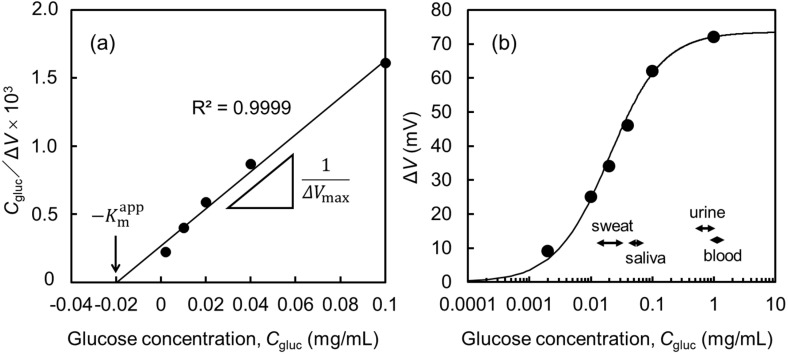
Hanes–Woolf plot (**a**) and a numerically calculated fitting curve (**b**) from using Equation (2). The solid circles are the experimental data read from Figure 6.

Figure 8a shows the Hanes–Woolf plot of the experimental data in Figure 6, where the straight line is fitted by a least squares method. The values of $\Delta V_{\max}$ and $K_{m}^{\mathrm{app}}$ estimated from the *x*-axis intercept and the slope of the straight line were 74 mV and 0.020 mg/mL, respectively. In Figure 8b, we show the experimental data (plots) and the numerically calculated curve by substituting $\Delta V_{\max}=74\;\mathrm{mV}$ and $K_{m}^{\mathrm{app}}=0.020\;\mathrm{mg}/\mathrm{mL}\;$ into Equation (2). It is indicated by the good agreement between the calculated curve and plots that our experimental data can be approximated by Equation (2). The lower and upper detection limits of this glucose sensor are roughly estimated to be 0.001 mg/mL and 0.5 mg/mL, respectively, by defining the effective operation range $\Delta V_{\mathrm{eff}}$ as $\Delta V_{\mathrm{eff}}=0.9\Delta V_{\max}$.

In Figure 8b, we also show the range of glucose concentrations in the blood, urine, saliva, and sweat in a healthy body [39]. Our EGFET-type glucose sensor can detect low concentrations such as those in the saliva and sweat. Although the glucose concentrations in the blood and urine are close to the upper detection limit, this can be settled by dilution and/or decreasing the GOD content in the SF membrane. For reference, the detection range of our EGFET-type glucose sensor is around two orders of magnitude lower than that of commercially available electrode-type portable glucose sensors.

In Table 1, we compared the detection range of our EGFET-type glucose sensor with those of reported FET-type glucose sensors using GOD. It is seen that many studies have been done from a wide variety of viewpoints, from high sensitivity to easy usability/cost efficiency, since glucose sensors are important devices for biomedical applications. In general, high sensitivity is achieved by employing nanostructures such as nanoparticles (NPs), nanorods (NRs), and nanowires (NWs) on the gate electrodes, although most of the reports do not refer to the endurance against repeated use and/or long-term stability. Compared with them, the sensitivity in this work is low but still high enough to detect the glucose in the sweat and saliva. The largest merit of our EGFET-type glucose sensor is that the extended gate electrode is fully composed of biocompatible materials with high reproducibility, cost-effectiveness, and easy handling. In addition, the employment of sputtered [30,31], sol-gel [32] and nanostructured TiO_2_ [33] might be likely to increase the cost-effectiveness and sensitivity.

Apart from sensitivity, interference by chemical species such as ascorbic acid and uric acid decreases the accuracy of the measured results for real body fluids, and it is known that the coating of the gate electrode by ion-selective membranes like Nafion is effective in minimizing the interference [13]. However, we have not studied the interference effect at present on our EGFET-type glucose sensors, and it is an important research task for us from now on.

### 3.4. Endurance of the Sensor

**Figure 9:**
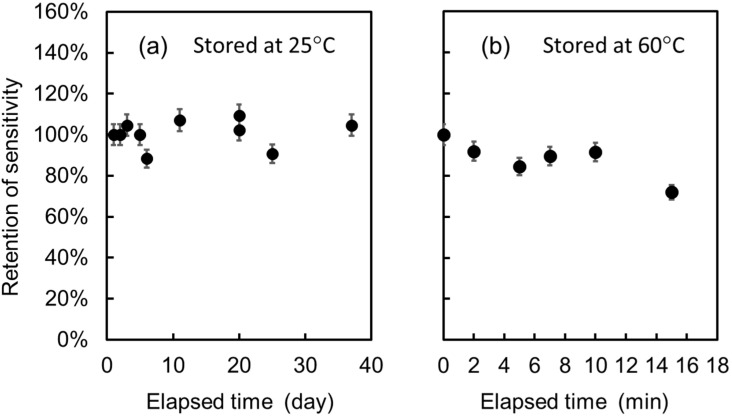
Retention characteristics of the EGFET-type glucose sensor for repeated measurements with long-term storage at 25 °C (**a**) and at 60 °C (**b**).

For practical usage, endurance against repeated use, long-term storage, and environmental conditions is required for biosensors. We examined our EGFET-type glucose sensor for endurance against repeated use and long-term storage as shown in Figure 9a, the sensitivity of which is represented by the Δ*V* for the change in glucose concentration from 0 to 1 mg/mL, and the retention was represented by Δ*V*/Δ*V*_0_ × 100, where Δ*V*_0_ is the Δ*V* at the initial measurement. In this experiment, the time required for each glucose measurement was about 1 h, and the extended gate electrode, after measurement, was removed from the MOSFET and thoroughly washed in deionized water and then stored in dried air (20% RH) at 25 °C. It is clearly indicated by the experimental data in Figure 9a that the extended gate electrode has stable sensitivity and can endure over 10 repeated operations and over 35 days of storage.

Since both GOD and SF are proteins, compositional and/or structural change will be induced at high temperatures. Especially, GOD is known to lose its enzymatic function due to the change in the steric structure at the active sites. In fact, free GOD starts to lose the activity at 40 °C and becomes inactive at 60 °C. However, it was reported that the GOD in the SF membrane came to possess higher stability [25,36] and keep full enzymatic activity even at 60 °C [25]. We also examined our extended gate electrode for endurance against heating as shown in Figure 9b. The notation for the vertical axis and the experimental conditions except the stored temperature are similar to those in Figure 9a. In this experiment, we stored the extended gate electrode after measurement at 60 °C in air.

Different from the case shown in Figure 9a, the sensitivity was easily decreased by storage at 60 °C; it decreased to 70% of the initial value after 15 min of storage. Similarly, although the detailed experimental data are not shown here, a decrease to 90% of the initial sensitivity was also observed when stored in deionized water at 60 °C for 30 min. While these data indicate the low heat-endurance of GOD against temperature, it is also shown that the GOD immobilization in the SF membrane really increases endurance against high temperatures, in agreement with previous reports [25,36]. From the viewpoint of practical usage, the increase in the heat tolerance from 40 °C to 60 °C is important for storage at around room temperature.

## 4. Conclusions

We prepared an EGFET-type glucose sensor by immobilizing GOD using an SF membrane on an ion-sensitive TiO_2_/Ti extended gate electrode. The GOD-containing SF membrane was formed on the TiO_2_/Ti extended gate electrode using a spin-coating method followed by an insolubilization process in an 80 vol % ethanol aqueous solution. The TiO_2_/Ti surface was treated in advance with a silane coupling agent to enhance the adhesivity of the SF membrane in aqueous solution.

The resulting EGFET-type glucose sensor had a lower detection limit of about 0.001 mg/mL, which is high enough to detect the glucose in the saliva and sweat. It was also demonstrated that the sensor can endure repeated operation at room temperature for over 30 days, with a high heat tolerance close to 60 °C. These successful results indicate that our EGFET-type glucose sensor using a GOD-containing SF membrane might be promising for application to reliable and cost-effective potentiometric glucose sensors for personal use and for the detection of other substrates by replacing GOD with specific enzymes.

## Figures and Tables

**Table 1:** Comparison of the presented EGFET with other FET-type glucose sensors.

Type	Structure	Detection Range (mg/mL)	Reference
ISFET(SGFET)	GOD/Diamond/Si	0.018–1.8	[11]
ISFET(SGFET)	GOD/Nafion/Pt NPs/Graphene/Glass	0.00009–0.18	[13]
ISFET(SGFET)	GOD/ZnO NRs/AlGaN/GaN	0.00000009–0.023	[14]
ISFET(SGFET)	GOD/InZnO/Glass	0.036–7.2	[15]
ISFET(SGFET)	SF-GOD/Graphene/SF	0.018–1.8	[22]
EGFET	GOD/ZnO NWs/Ag wires	0.00018–0.018	[18]
EGFET	GOD/SnO_2_/ITO/PET	1–3	[19]
EGFET	GOD/ZnO NRs/ITO/Glass	0.0036–0.18	[20]
EGFET	GOD/TiO_2_/Ti	0.0045–0.45	[21]
EGFET	SF-GOD/TiO_2_/Ti	0.001–0.5	This work
